# Expression of *ywhaz* and gene regulation network in hepatocellular carcinoma

**DOI:** 10.3892/ol.2020.11996

**Published:** 2020-08-20

**Authors:** Yi Lin, Ling Sun, Xiaolei Ye

Oncol Lett 19: 3971-3981, 2020; DOI: 10.3892/ol.2020.11481

Owing to an error in the production process, the gene encoding the adaptor protein 14-3-3ζ, *ywhaz*, was spelt incorrectly as “yhwaz” throughout the entirety of this paper. The corrected title for this article is featured above; this will be corrected accordingly in PubMed. The incorrect spelling for this gene was featured a total of 104 times in the article; in view of the large number of corrections, an updated version of the PDF has also been upoaded to PubMed and to the Journal's website. We regret that this error was introduced into the article, and apologize to the authors for any inconvenience caused.

